# Stress and dental caries among dental students at Pharos university in Alexandria

**DOI:** 10.1186/s12903-025-06873-8

**Published:** 2025-09-17

**Authors:** Maha Ibrahim Adel, Inas Karawia

**Affiliations:** Dental Public Health and Preventive Dentistry Department, Faculty of Dentistry, Pharos University in Alexandria, Alexandra, Egypt

**Keywords:** Psychological stress, Human dental caries, Dental students

## Abstract

**Introduction:**

Stress may increase the susceptibility to dental caries in humans. This study aimed to assess stress levels among dental students at Pharos University in Alexandria and explore their relationship with dental caries across different academic years.

**Materials and methods:**

This study was an analytical cross-sectional design, conducted among 80 first- and fifth-grade dental students at Pharos University. The participants were asked to sign an informed consent form before the study began. DESQ was used to measure the stress of dental students. Dental caries was evaluated using DMFT.

**Results:**

The results found that being a female student significantly increased the self-efficacy belief category of stress (B = 1.3, *P* = 0.013 ). Gender did not affect faculty and administration, workload, patient treatment, clinical training, performance pressure, other/personal factors, as well as total stress (*P* > 0.05). Being a student in grade 5 increased the level of all categories of stress significantly when compared with students in grade 1 (B = 3.5, *P* = 0.000, B = 9.8, *P* = 0.000, B = 4.9, *P* = 0.000, B = 13.2, *P* = 0.000, B = 0.58, *P* = 0.017, B = 9.9, *P* = 0.000, B = 5.1, *P* = 0.000 and B = 23.6, *P* = 0.000 respectively). When examining the relation between stress and dental caries, it was found that self-efficacy beliefs (*r* = 0.2, *P* = 0.076) and clinical training (*r* = 0.081, *P* = 0.475) had no relation with dental caries. However, faculty and administration (*r* = 0.4, *P* = 0.001) workload (*r* = 0.5, *P* = 0.000), patient treatment (*r* = 0.43, *P* = 0.000), performance pressure (*r* = 0.3, *P* = 0.007 ) and other/personal factors (*r* = 0.4, *p *= 0.001) had a significant direct correlation with dental caries among examined dental students, and this is lead to a high significant direct relation between the total stress and dental caries (*r* = 0.4, *P* = 0.003).

**Conclusions:**

Environmental stress among dental students had a significant positive effect on dental caries.

## Introduction

The decay of teeth caused by bacteria and dietary factors is a prevalent oral health problem globally. This decay leads to the destruction of tooth structure [1]. The daily diet plays a significant role in influencing the development and advancement of the disease due to carbohydrate consumption. Additionally, research studies have indicated that the prevalence of tooth decay is linked to behavioral, social, economic, and clinical factors [2].

Stress can increase susceptibility to dental caries through four potential mechanisms: it can impact the immune system, leading to heightened vulnerability to dental caries [1]. by reducing the host’s ability to fight against cariogenic microorganisms [3] and lowering salivary production, which will result in less clearance [4, 5] unhealthy emotional eating patterns, which are then followed by being eating more often and consuming a diet high in sugar [6, 7], and ultimately, poor self-care habit implementation [8].

All efforts to comprehend the impact of social and physiological variables on oral and overall health are based on the crucial idea of stress [9]. There is a definition that uses the phrase “an inharmonious fit between the person and the environment, one in which the person’s resources are used up or exceeded and subject to effort or what is called in normal speech more or less struggle, usually in complex ways, to be able to endure this,” to describe stress. This is why people may attempt to eliminate either the stressor or the stress itself when they begin to perceive an event as something more than they can handle and utilize resources for its management that are available to them and only at this point, do they perceive the stress there that may cause diseases [10].

Dental students face numerous challenges in their daily work. Stress is one of the issues encountered. The fear of academic grades, frequent exams, an overwhelming workload, meeting course completion deadlines, socioeconomic pressures, relationships with peers and teachers, and indications of psychological morbidity are just a few of the work-related stressors that dental students, both undergraduate and graduate, encounter. Professional training which includes academic and clinical practice generates substantial stress resulting in inefficiency causing failure to achieve maximum productivity [11, 12].

A study in Saudi Arabia (2017) using the DASS-21 showed that about two-thirds of 277 undergraduate dental students had high stress, leading the authors to call for targeted preventive measures [13]. A similar investigation in India (2021) with the Dental Environment Stress Questionnaire (DESQ) found higher stress in preclinical than clinical students and recommended curriculum-integrated stress-reduction strategies [14]. Likewise, a Mexican study (2020) using the 10-item Perceived Stress Scale (PSS) reported that over 60% of dental students experienced high stress, underscoring the need for early identification and support by educators [15].

International data consistently link higher academic stress with poorer oral-health outcomes. In Bangalore, India, Jain et al. (2014) found that pre-university students with elevated perceived-stress scores had a significantly greater caries experience than their less-stressed peers [16]. Another study (2012) followed Mexican students for six months and showed that those reporting moderate-to-high academic stress, especially when coupled with low salivary flow were two to three-times more likely to develop new carious lesions [17]. Complementing these findings, a cross-sectional survey in North-West Russia that applied the PSS-10 and DMF index reported that dental students not only scored higher on stress than medical students but also had a greater number of decayed teeth, underscoring a probable stress–caries link in yet another setting. Taken together, these studies support the view that elevated academic stress is an independent risk factor for dental caries across diverse populations [18]. In 2019, a study in Iraq documented a positive correlation between dental caries and high levels of dental environment stress among dental students [19]. 

In Egypt (2012), a study was carried out to recognize the sources of stress among dental students enrolled at Pharos University in Alexandria (PUA) – Egypt. It was found that mid-senior students demonstrated higher stress in comparison to junior students, recommended that. In order for students to manage and overcome the challenges and overloads they encountered while studying, the educational system should address potential stressors for them through stress management programs [20].

Although international studies in India, Mexico Russia and Iraq have linked perceived stress to higher DMFT scores among university dental students, To the best of our knowledge, no previous study has examined the association between stress and dental caries among Egyptian dental students. Because Egypt differs markedly in sugar-consumption patterns, dental-care utilization and educational stressors, investigating this cohort addresses an important geographic and contextual evidence gap.

For this limitation, this study aimed to evaluate the stress among dental students in Pharos University in Alexandria and show its relation with dental caries in different academic years. Our null hypothesis was there is no difference in stress severity among students in different academic years, and there is no relation between stress and dental caries experience.

## Materials and methods

### Study design and setting

The current study was an analytical cross-sectional study among first and fifth-grade dental students at Pharos University. The study was conducted after the approval of the ethics committee at Pharos University under registration number (UREAC-02-3-171). At the beginning of the Google Form, a cover letter with information to participants about the study’s purpose, confirming its voluntary and anonymous nature. Participants were also provided with an estimate of the time required to complete the form. Before filling out the questionnaire, written informed consent was obtained from all participants. The study adhered to the STROBE reporting guidelines for observational studies, ensuring a high standard of methodological rigor and transparency.

### Sample size and sampling technique

The sample size was calculated using G power software by taking 0.27 as an effect size from a previous study examining the relation between stress and salivary flow among dental students [21] and Alpha error = 0.05, Power = 0.80, and Confidence interval at 95% to be 78 rounded to 80 dental students. The participation of undergraduate dental students of grades one and five included voluntary, in case of the presence of any systemic disease; chemotherapy or radiation treatment of the head and neck; the continuous use of any medication; the presence of fixed or removable dental appliances; students with poor oral hygiene and pregnant female students were excluded from the study. The survey was anonymous, ensuring participant confidentiality. Written informed consent was obtained before participants completed the self-structured questionnaire.

### Data collection tool

Dental environment stress was recorded using a validated previously published self-recorded questionnaire for all dental students [22] during the 1 st month of the second academic term. The dental environment stress questionnaire (DESQ) is a specific scale designed to measure the stress of dental students it consists of 38 questions, and for each question, 5 scales from 0 to 4 (not applicable, not stressful, moderately stressful, stressful, very stressful), so the students have to answer for each event by choosing one of these alternatives. The 38 questions were categorized into 7 categories; self-efficacy beliefs, faculty and administration, workload, patient treatment, clinical training, performance pressure and personal factors.

Oral caries experience was evaluated using DMFT; D = Decay, M = Missed, F = Filled surface. Students were examined on the dental chair using WHO probes and mirrors [23].

### Statistical analysis

The data was collected, coded, and analyzed using SPSS version 25 software. 5% was used to be the significance level of the achieved results. Frequency and percent were used for categorical variables, and Mean ± SD was used for numerical data presentation. Linear regression was used to evaluate the relation between students’ demographics and stress mean values. Correlation was done to show the relation between stress and dental caries among students.

## Results

Approximately half of the participants were male (47.5%), while the remaining were female (52.5%). As well the sample was divided equally to be 50% for both grade 1 and grade 5 students (Table 1).

**Table 1:** Distribution of the participants by their demographic data

	No.	%
Gender		
Male	38	47.5
Female	42	52.5
Academic year		
Grade 1	40	50%
Grade 5	40	50%
Age		
mean ± SD	20.15 ± 2.0	
Range	17–23	

Table 2 shows the relation between independent variables and stress using regression analysis. Being female or a fifth-year student significantly increased stress levels in the self-efficacy belief category. (B = 1.3, *P* = 0.013 and B = 3.5, *P* = 0.000 respectively). Gender variable did not affect Faculty and administration, workload, patient treatment, clinical training, performance pressure, other/personal factors as well as total stress (B = 0.4, *P* = 0.57, B = 0.06, *P* = 0.85, B = 0.4, *P* = 0.30, B = 0.07, *P* = 0.78, B = 0.93, *P* = 0.20, B = 0.17, *P* = 0.69 and B = 2.9, *P* = 0.22). It was found that on the other hand, being a student in grade 5 increased the level of all categories of stress significantly when compared with students in grade 1 (B = 3.5, *P* = 0.000, B = 9.8, *P* = 0.000, B = 4.9, *P* = 0.000, B = 13.2, *P* = 0.000, B = 0.58, *P* = 0.017, B = 9.9, *P* = 0.000, B = 5.1, *P* = 0.000 and B = 23.6, *P* = 0.000 respectively).

**Table 2:** Relation between independent variables and stress among participants

	*R* ^2^	Predictor	Mean ± SD	B	*P* value
**Self-Efficacy Beliefs**	43.2	**Gender**			
		Male ^Ref^		1.3	0.013*
		Female			
		**Academic year**			
		Grade 1^Ref^		3.5	0.000*
		Grade 5			
**Faculty and Administration**	84.8	**Gender**			
		Male ^Ref^		0.4	0.57
		Female			
		**Academic year**			
		Grade 1^Ref^		9.8	0.000*
		Grade 5			
**Workload**	63.7	**Gender**			
		Male ^Ref^		0.06	0.85
		Female			
		**Academic year**			
		Grade 1^Ref^		4.9	0.000*
		Grade 5			
**Patient treatment**	93.9	**Gender**			
		Male ^Ref^		0.4	0.30
		Female			
		**Academic year**			
		Grade 1^Ref^		13.2	0.000*
		Grade 5			
**Clinical training**	7.2	**Gender**			
		Male ^Ref^		0.07	0.78
		Female			
		**Academic year**			
		Grade 1^Ref^		0.58	0.017*
		Grade 5			
**Performance pressure**	71.2	**Gender**			
		Male ^Ref^		0.93	0.20
		Female			
		**Academic year**			
		Grade 1^Ref^		9.9	0.000*
		Grade 5			
**Other/personal factors**	**66.1**	**Gender**			
		Male ^Ref^		0.17	0.69
		Female			
		**Academic year**			
		Grade 1^Ref^		5.1	0.000*
		Grade 5			
**Total Stress**	83.5	**Gender**			
		Male^Ref^	60.8 ± 26.4	2.9	0.22
		Female	61.3 ± 25.8		
		**Academic year**			
		Grade 1^Ref^	61.4 ± 25.8	23.6	0.000*
		Grade 5	84.7 ± 13.5		

*: Significance level at P value ≤ 0.05

^Ref^: Reference variable

When the relation between stress and dental caries was examined (Table 3), it was found that self-efficacy beliefs (*r* = 0.2, *P* = 0.076) and clinical training (*r* = 0.081, *P* = 0.475) had no relation with dental caries. However, faculty and administration (*r* = 0.4, *P* = 0.001) workload (*r* = 0.5, *P* = 0.000), patient treatment (*r* = 0.43, *P* = 0.000), performance pressure (*r* = 0.3 , *P* = 0.007) and other/personal factors (*r *= 0.4, *P *= 0.001) had a significant direct correlation with dental caries among examined dental students, and this is lead to a high significant direct relation between the total stress and dental caries (*r* = 0.4, *P* = 0.003).

**Table 3:** Relation between stress and dental caries among participants

	DMFT	
Self-efficacy beliefs	r	0.2
	P	0.076
Faculty and administration	r	**0.4**
	P	0.001*
Workload	r	**0.5**
	P	0.000*
Patient treatment	r	**0.43**
	P	0.000*
Clinical training	r	0.081
	P	0.475
Performance pressure	r	**0.3**
	P	0.007*
Other/personal factors	r	**0.4**
	P	0.001*
Total Stress	r	**0.4**
	P	0.003*

*: Significance level at *P* value ≤ 0.05

r: correlation coefficient

## Discussion

Dental school is a highly stressful environment, with dental students experiencing more stress than both medical students and the general population. Similarly, dental students are expected to perform well in academics and clinically during their studies that makes this process very stressful [24]. 

The present study shows that in healthy students aged 17–23 years gender variable did not affect the level of stress among students (B = 2.9, *P* = 0.22) and that stress was significantly higher (*P* = 0.000) among fifth-grade students. This was in agreement with a study conducted in India in 2011, the authors found that final year dental students presented with higher stress scores [25].

When the relation between stress and dental caries was examined, it was found that self-efficacy beliefs (*r* = 0.2, *P* = 0.076) and clinical training (*r* = 0.081, *P* = 0.475) had no relation with dental caries. This result was in Line with that reported by Myrvold B. 2017, who found that responses to these 2 categories of the DESQ were not related to dental caries [26].

However, faculty and administration (*r* = 0.4, *P* = 0.001) scored high denoting a significant correlation which is in agreement with Ersan N. et al. 2018. Although, in a recent study in Jordan Al Amoush R, 2024 reported that this factor scored the lowest in comparison with other stressor factors [27]. These differences may stem from variations in staff, institutional policies, and other factors between universities. At Pharos University, 5th grad dental students are required to complete a full-mouth comprehensive care case as part of their graduation project, which demands more clinical time, and increase the students’ fear from receiving criticism from their supervisors, potentially leading to increased stress from faculty and administration. This heightened stress could, in turn, be linked to an increase in dental caries.

The present study revealed that workload was among the most common stressors (*r* = 0.5, *P* = 0.000), which was reported also as a high stressor in a study by Olson H. et al. 2019 of dental and oral health therapy students [28]. It was believed that workload was the primary stressor perceived by dental students, being much greater than war-related stress, which ranked as the second highest source of perceived stress in Yemen. This is largely due to their demanding schedules, filled with lectures, laboratory work, and clinical sessions, leaving little time for relaxation [29]. This may explain why workload had the highest correlation with dental caries than the remaining stressor domains.

Regarding patient treatment as a stressor, the present study shows a significant direct correlation with dental caries among examined dental students (*r* = 0.43, *P* = 0.000). Because fifth-year procedures, during completing the requirements of their comprehensive case care, like endodontic treatment of molar, and fixed and removable prosthodontics preparations, need more time to complete, grade 5 students ranked the absence of patient follow-up as the biggest stressor. Additionally, patients’ variables were crucial, patient commitment-related aspects made students anxious about it and made them more stressed out overall. This result was in agreement with another studies [30, 31]. The results of the current study were also supported by the observation of Elagra et al. that border molding, final impression, and jaw relation records were the three procedures in complete denture delivery that stressed dental students more than any other. The difficulty of these procedures was the most commonly reported factor that causes stress [32].

However, tests, grades, fear of failing, and other performance pressure components of DESQ (*r* = 0.4, *P* = 0.001) were listed as stressors for fifth-grade dentistry students. Notably, our findings were corroborated by a study of dentistry students at the University of Jordan in 2016 [33] and another study by Smolana et al. 2022 who documented performance pressure and study conditions as the 2 biggest stressors which coincide with general conclusions from the literature review and appears to be natural for students [34].

There was a strong direct association between dental caries and other/personal variables of the DESQ (*r* = 0.4, *P* = 0.003) Similarly, other studies identified that personal life, and professional identity, are correlated positively with depression, anxiety, and general and perceived stress among final year dental students [35, 36].

Stress decreases salivary flow rate, and stress drugs may produce atrophy in major salivary glands, which also alters salivary composition and volume. Corticosteroids have been shown to cause hypoplasia, which increases susceptibility to dental caries [37]. Also, stress affects immunity and can weaken the host’s defense against cariogenic bacteria.

A study conducted in 2020, aimed to evaluate the relation between environmental stress and dental caries among Iraqi dental students. Students were classified as mild, moderate and severe stress according to DESQ. It was found that there is a positive relationship between stress and dental caries and the researcher related these findings to the presence of nitrous oxide in the saliva, which is a biomarker of stress [19]. The salivary bacterial count increased with the increased formation of nitrous oxide [38].

In our investigation, no relationship was found between clinical training-related stress or self-efficacy beliefs and the experience of dental caries among dental students. These results could be explained by several reasons, such as relatively similar levels of clinical exposure among participants, or the generally high self-efficacy that many dental students possess [39]. It is also important to consider that dental caries is a multifactorial disease [40], and psychological stressors may influence it indirectly by affecting daily habits like diet or oral hygiene. Although a direct link was not detected, both clinical training and self-efficacy remain critical elements in the dental education process. Supporting students in these areas is still essential for promoting academic success and overall well-being.

The study’s cross-sectional design limits the ability to understand the timing of stress exposure, students’ stress responses may indicate recent experiences and dental caries develops as a chronic, cumulative condition. Additionally, the current study did not measure dental caries activity or dietary habits, making it difficult to determine the temporal relationship between stress and dental caries and, consequently, to establish causality. Another significant limitation is the dependence on a convenience sample, which may not represent the broader population. One more limitation of this study is the absence of a control group outside the dental education context. Without such a group, it is difficult to determine whether the observed associations are unique to dental students or reflect broader student stress patterns. Future research is encouraged to include such comparison groups to strengthen the generalizability of findings. Furthermore, using self-reported stress scales introduces the possibility of inaccurate responses, as participants may provide incorrect information rather than objective observations.

## Conclusion

Fifth-year dental students reported higher stress levels than first-year students, with workload, faculty and administration, patient treatment, and performance pressure identified as key stressors, likely due to the intensive curriculum. Other factors, such as grades, fear of failure, and limited leisure time, contributed to performance pressure. The transition from pre-clinical to clinical phases was also stressful. Environmental stress was found to significantly increase dental caries, while factors like gender, clinical training, and self-efficacy had no impact.

### Recommendations

Longitudinal studies are needed to validate the relationship between stress and dental caries. Given the lack of prior research linking academic stress to dental caries, further studies are needed to examine how each clinical component influences students’ stress levels and explore stress-reduction strategies for dental students. Faculty management must take proactive measures to address potential stressors and help students manage the challenges of their education. The development of dental curricula and working environments should consider both academic and non-academic stress sources, with an emphasis on adopting creative and engaging teaching methods. Assessing personality traits and measuring stress levels before admission can help identify the true increase in stress during dental education. Implementing these changes will reduce stress, supporting students’ academic success and professional development. Additionally, periodic screening for oral diseases among dental students is essential for early diagnosis and treatment. Also, including a control group of non-dental or pre-dental students could have helped isolate the specific impact of dental education-related stress on caries experience.

#### Practical implications

These findings highlight the need for targeted stress-reduction interventions within dental curricula, particularly for senior students transitioning to clinical training. Universities should consider:

- Implementing stress management programs (e.g., time management workshops, mindfulness training, or counseling services) to help students cope with academic and clinical demands.
- Reviewing the clinical curriculum and workload to identify ways to reduce unnecessary pressure without compromising educational quality.
- Promoting oral health awareness among students, emphasizing how chronic stress may increase caries risk and the importance of preventive dental care for themselves as future professionals.
- Conducting regular stress assessments to monitor student well-being and inform institutional changes.

## Data Availability

No datasets were generated or analysed during the current study.
